# Allogeneic Mesenchymal Stromal Cell Injection to Alleviate Ischemic Heart Failure Following Arterial Switch Operation

**DOI:** 10.1016/j.jaccas.2021.02.039

**Published:** 2021-05

**Authors:** Filippo Rapetto, Demetris Taliotis, Qiang Chen, Iakovos Ttofi, Dominga Iacobazzi, Paolo Madeddu, Serban C. Stoica, Ben Weil, Mark W. Lowdell, Massimo Caputo

**Affiliations:** a Department of Cardiac Surgery, Bristol Heart Institute and Bristol Royal Hospital for Children, Bristol, United Kingdom; b Translational Health Sciences, University of Bristol, Bristol, United Kingdom; c Department of Cardiology, Bristol Heart Institute and Bristol Royal Hospital for Children, Bristol, United Kingdom; d Centre for Cell, Gene & Tissue Therapeutics, Royal Free London NHS Foundation Trust, London, United Kingdom

**Keywords:** acute heart failure, coronary vessel anomaly, transposition of the great arteries, CHD, congenital heart disease, FAC, fractional area change, GLS, global longitudinal strain, LCA, left coronary artery, LV, left ventricular, MSC, mesenchymal stromal cell

## Abstract

Cell therapy is a promising tool to prevent and treat heart failure in congenital heart disease. We report the first case of intramyocardial injection of allogeneic mesenchymal stromal cells as rescue therapy in a neonate with ischemic heart failure following arterial switch procedure for isolated transposition of the great arteries. (**Level of Difficulty: Advanced.**)

## Central Illustration

**Figure:**
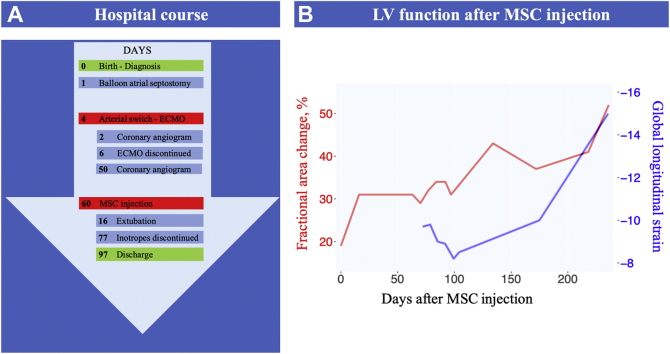

## History of Presentation

A 3.7-kg male neonate underwent an arterial switch operation for isolated transposition of the great arteries on day 4 of life. Intraoperatively, the left coronary artery (LCA) was found to have a long intramural course. The patient developed acute left ventricular (LV) dysfunction after separation from cardiopulmonary bypass. Refashioning of the LCA button was attempted, supplemented by a coronary artery bypass graft (left internal mammary artery to left anterior descending coronary artery). Venoarterial extracorporeal membrane oxygenation was instituted before transferring the patient to the intensive care unit and successfully discontinued on post-operative day 6.

**Learning Objectives**

• To highlight an uncommon and potentially fatal complication of the arterial switch operation.
• To illustrate a novel promising application of cell therapy in patients with CHD.

## Medical History

The patient had been born at 39 + 2 weeks of gestation by normal vaginal delivery and had developed oxygen desaturation (50% despite fraction of inspired oxygen of 100%) and hypotension (mean arterial blood pressure of 25 mm Hg) at 6 h of life. He had then been diagnosed with transposition of the great arteries with intact ventricular septum. Percutaneous balloon atrial septostomy had been undertaken and surgery had been planned.

## Differential Diagnosis

Acute intraoperative heart failure during the arterial switch operation is virtually always caused by myocardial ischemia related to suboptimal coronary transfer. Severe pulmonary vascular disease is an uncommon cause of right ventricular failure, which was not the case in this patient, who had isolated LV dysfunction.

## Investigations

**Figure 1:**
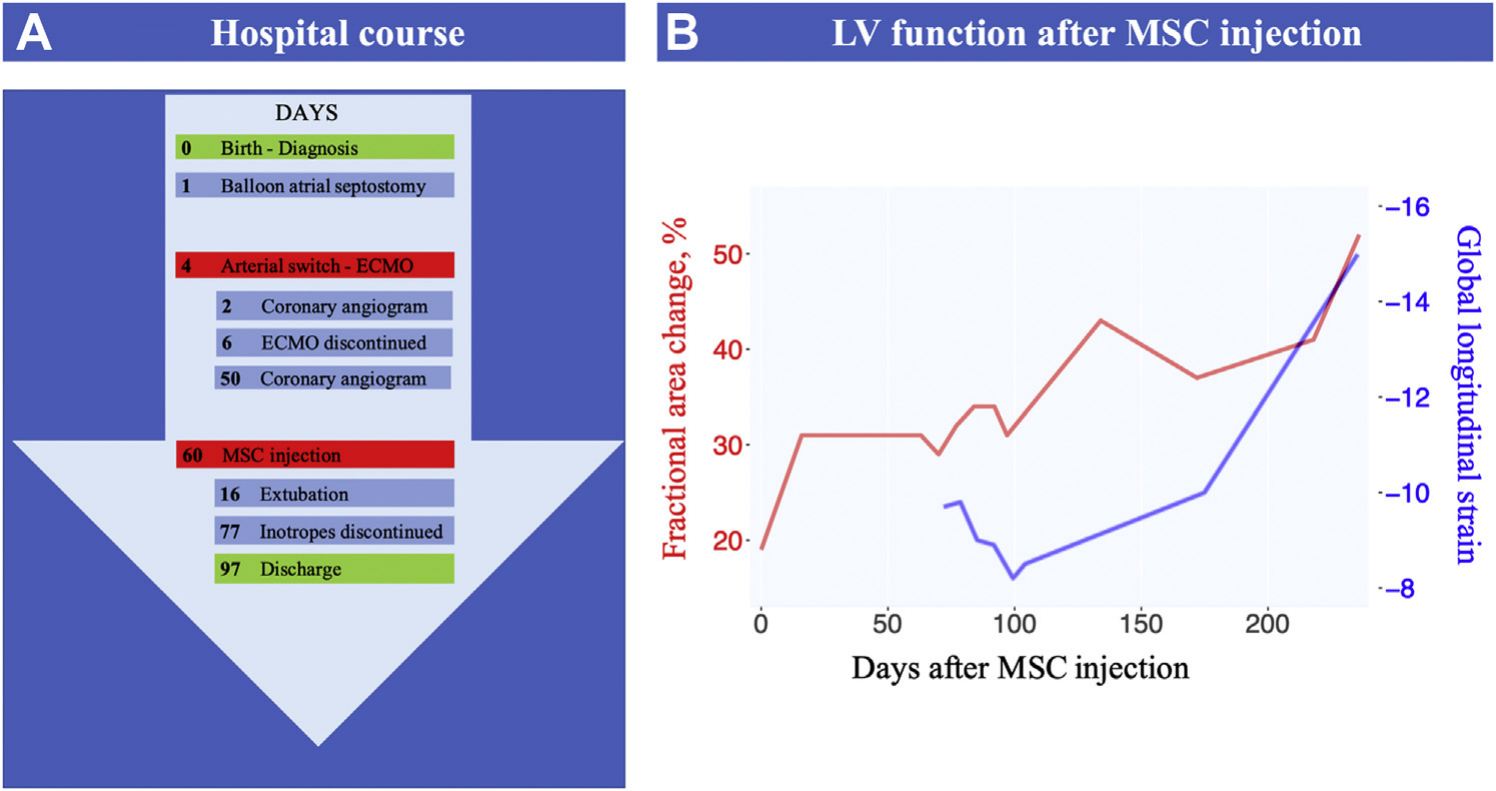
Clinical Events and LV Function **(A)** Hospital course and **(B)** evolution of left ventricular (LV) function after intramyocardial mesenchymal stromal cell (MSC) injection to palliate ischemic heart failure following arterial switch operation. The **blue line** on the plot represents LV global longitudinal strain, and the **red line** represents LV fractional area change measured by transthoracic echocardiography. ECMO = extracorporeal membrane oxygenation.

Coronary angiography on post-operative day 2 showed LCA and internal mammary artery occlusion. Angiography was repeated on post-operative day 50, confirming the LCA occlusion and demonstrating only modest collateral circulation from the right coronary artery (Video 1). Serial transthoracic echocardiograms were obtained post-operatively to assess the evolution of LV function (Figure 1).

## Management

Over the first 2 post-operative months, the patient developed inotrope- and ventilator-dependent LV systolic dysfunction, with failure to thrive and echocardiographic signs of anterolateral LV ischemic injury, including anterior wall akinesia, a small anterior wall LV aneurysm, and basal to mid anterior wall hyperechogenicity.

The patient was deemed suitable for intramyocardial injection of allogeneic mesenchymal stromal cells (MSCs). The treatment was approved on a compassionate basis by the University Hospital Bristol and Weston NHS Foundation Trust.

The Centre for Cell, Gene and Tissue Therapeutics (Royal Free Hospital, University College London) provided pharmaceutical-grade MSCs. The MSCs were derived from a single umbilical cord, supplied by the Anthony Nolan Cord Blood Bank from a registered volunteer cord blood donor procured under Human Tissue Authority license. Umbilical cord tissue-derived MSCs were isolated by plastic adherence, expanded, and cryopreserved until the day of administration; they were tested for purity (following International Society for Cellular Therapy criteria [1]), sterility, and endotoxin. The drug product was prescribed, manufactured, and supplied as an unlicensed medicinal product (special) in accordance with U.K. law.

MSC injection was undertaken on post-operative day 60. The anterolateral LV wall was accessed via a small left anterior thoracotomy. The target dose of 3 million cells/kg body weight was administered with 4 separate subepicardial injections following a technique previously used in patients with hypoplastic left heart syndrome (HLHS) (2). The patient was extubated and weaned from all intravenous inotropes 16 and 77 days after MSC injection, respectively. He was discharged home 97 days after the procedure on oral heart failure therapy and remained clinically stable.

LV ejection fraction was 58% after extracorporeal membrane oxygenation discontinuation and had deteriorated to 23% at MSC injection despite inotropic support. After MSC injection, LV function was monitored mainly with LV fractional area change and LV global longitudinal strain. Videos 2 and 3 show corresponding views of pre-injection and pre-discharge transthoracic echocardiograms, respectively. Clinical course and LV function changes after MSC injection are summarized in Figure 1.

## Discussion

Coronary artery abnormalities remain a surgical challenge when performing the arterial switch operation, increasing surgical morbidity and mortality because of myocardial ischemic injury (3).

Cell therapy can be delivered in different ways according to 3 main parameters. First, a cell type must be selected. To date, umbilical cord cells, peripheral blood or bone marrow–derived mononuclear cells, cardiac stem cells, and MSCs have been used clinically in congenital heart disease (CHD). MSCs can be easily isolated from different tissues and expanded in vitro, have intrinsic differentiation potential, and show marked modulatory properties that are thought to enhance the host’s regenerative processes, which is believed to be more important than the MSC expansion itself. It is known that MSCs can stimulate recovery from cardiac injury through paracrine activity, promoting reduction of fibrosis, neovascularization, immunomodulation, and stimulation of endogenous tissue regeneration (4).

Second, the selected cells can be of autologous or allogeneic origin. The main theoretical advantage of autologous cells is their immunologic compatibility; nevertheless, there is little evidence that allogeneic MSCs, which express low levels of major histocompatibility complex class I and lack major histocompatibility complex class II antigens, induce alloantibodies. In contrast, the immediate availability of allogeneic cells renders them more feasible in an urgent setting. Moreover, the recent POSEIDON-DCM (Percutaneous Stem Cell Injection Delivery Effects on Neomyogenesis in Dilated Cardiomyopathy) randomized trial showed superiority of allogeneic MSCs over autologous MSCs in an adult cohort of patients with dilated cardiomyopathy, possibly due to better modulatory properties (5).

Third, the route of delivery must be chosen; to date, intramyocardial and intracoronary administration have been undertaken clinically (6).

Cell therapy has been used in preclinical models of CHD and showed initial promising results in patients with hypoplastic left heart syndrome (2,7, 8, 9). To the best of our knowledge, this is the first report of successful use of allogeneic MSCs for inotrope- and ventilator-dependent ischemic heart failure following arterial switch operation. Our choice to use allogeneic cells was driven mainly by the urgent scenario; intramyocardial delivery was dictated by the occlusion of the LCA.

MSC injection does not resolve the culprit coronary lesion, and the exact mechanism of action in this particular patient cannot be precisely demonstrated. However, we observed a correlation between MSC injection and the patient’s clinical improvement; the rationale behind this specific application is that boosts of MSCs may aid the ischemic myocardium, thanks to their acknowledged proangiogenic activity.

## Follow-Up

One year after arterial switch, the patient remains on oral heart failure therapy with no clinical signs of ongoing myocardial ischemia, and he has been growing along the ninth weight centile. LV function is mildly impaired (Figure 1).

## Conclusions

Cell therapy is an emerging therapeutic strategy in patients with CHD. Its medium- and long-term results are not known, and further studies are needed to delineate potential candidates and outcomes in the pediatric population.

## Funding Support and Author Disclosures

This work was funded by the British Heart Foundation, the Sir Jules Thorn Award for Biomedical Research, and the Enid Linder Foundation. The authors have reported that they have no relationships relevant to the contents of this paper to disclose.

## Appendix

Supplemental Video 1 Coronary Angiogram Demonstrating Normal Right Coronary Artery Flow With Collateral Circulation to Occluded Left Coronary Artery

Supplemental Video 2 Echocardiographic Views Showing LV Contractility With Anterolateral Echogenicity, LV Anterior Aneurysm, and Trivial Mitral Regurgitation Immediately Before Cell Therapy

Supplemental Video 3 Echocardiographic Views Corresponding to Video 2 Showing Slight Improvement in LV Function With Unmodified LV Aneurysm and Mitral Regurgitation at Discharge
